# Odontogenic myxofibroma: A concise review of the literature with emphasis on the surgical approach

**DOI:** 10.4317/medoral.19842

**Published:** 2014-08-17

**Authors:** Marco Meleti, Ilaria Giovannacci, Domenico Corradi, Maddalena Manfredi, Elisabetta Merigo, Mauro Bonanini, Paolo Vescovi

**Affiliations:** 1 DDS, PhD, Consultant Professor, Department of Biomedical, Biotechnological and Translational Sciences, Centre of Oral Laser Surgery and Oral Pathology, Dental School, University of Parma, Italy; 2 DDS, Resident, Department of Biomedical, Biotechnological and Translational Sciences, Centre of Oral Laser Surgery and Oral Pathology, Dental School, University of Parma, Italy; 3 MD Associate Professor, Department of Biomedical, Biotechnological and Translational Sciences, Section of Human Pathology and Histopathology, University of Parma, Italy; 4 DDS, PhD, Assistant Professor, Department of Biomedical, Biotechnological and Translational Sciences, Centre of Oral Laser Surgery and Oral Pathology, Dental School, University of Parma, Italy; 5 DDS, Msci, Consultant Professor, Department of Biomedical, Biotechnological and Translational Sciences, Centre of Oral Laser Surgery and Oral Pathology, Dental School, University of Parma, Italy; 6 MD, DDS, Full Professor, Department of Biomedical, Biotechnological and Traslational Sciences, Centre of Oral Laser Surgery and Oral Pathology, Dental School, University of Parma, Italy; 7 DDS, Msci, Associate Professor, Department of Biomedical, Biotechnological and Translational Sciences, Centre of Oral Laser Surgery and Oral Pathology, Dental School, University of Parma, Italy

## Abstract

Purpose: The aim of this work is to report a review of the literature concerning epidemiology, clinical and radiographic features as well as treatment of odontogenic myxofibroma (MF). Methods: The PubMed database was searched using the following keywords: “odontogenic myxofibroma”, “odontogenic fibromyxoma”, “myxofibroma of the jaw” and “fibromyxoma of the jaw”. Results: Fifteen articles reporting the experience with 24 patients were identified. Male/female ratio was 1:1.4 and the average age was 29.5 years. The most frequent location was the mandible. In 66.7% of the cases the radiographic appearance was a multilocular radiolucency. Swelling was observed in 13 patients (92.86%), varying degrees of pain in 5 (35.71%) and paresthesia in only one patient (7.14%). Six out of 24 patients (26.09%) were treated with radical surgery and 17 (73.91%) with a conservative approach. In two out of 21 cases (9.52%) a recurrence was reported. Conclusions: MF is an extremely rare tumor and no agreement exists on the causes of its development. According to the present review, the choice of treatment should depend on variables such as localization, presence of a primary or of a recurrent lesion, age, general medical conditions and aesthetic needs of the patient.

** Key words:**Odontogenic myxofibroma, myxofibroma of the jaw, odontogenic tumors, oral surgery, oral pathology.

## Introduction

Myxofibroma (MF) of the jaws is a rare, benign, odontogenic tumor of possible mesenchimal origin firstly described by Virchow in 1863 (1).

MFs are variants of odontogenic myxomas that contain considerable amounts of collagen fibers dispersed within a myxoid stroma (2). Myxomas represent some 2.3% to 17.7% of all odontogenic tumors, MFs representing a small number of all myxomas (3). To the best of our knowledge, only 24 specific cases of MFs have been reported and described in details in the English literature since 1950. Incidence of such a tumor is approximately 0.05 new cases per million population per year (4).

MFs occur more frequently between the ages of 10 and 30 years (5). These tumors are more common in women and located mainly in the mandible, particularly in the posterior region (6).

Smaller lesions are usually asymptomatic and discovered during routine radiographic examinations, while large lesions are often associated with painless jaw expansion and possible perforation of the cortical plate. Facial deformity as well as involvement of the maxillary sinus have rarely been reported (6,7).

Histologically MFs consist of large amount of intercellular substance rich in acid mucopolysaccharides and made up of loose myxomatous connective tissue, fibroblasts and myofibroblasts. Patches of trabeculae of woven bone and capillaries are usually dispersed in the lesion (8).

Pathogenesis of MF is unknown. The microscopic similarity with mesenchymal tissue may lead to hypothesize that MFs derive from an abnormal development of the pulp organ during tooth embriogenesis.

Radiographically, MF appears as a unilocular or multilocular radiolucency with irregular or scalloped margins which may displace or cause resorption of the roots of adjacent teeth (9).

No agreement exists on the best management strategy. Treatment options vary from a conservative approach, consisting in the enucleation of the lesion and courettage of the cavity, to radical surgery. Some Authors suggest to extend surgical margins at least 1.5 cm around the neoplasm (6). Tumor excision may be associated to the extraction of the possibly related teeth. The conservative approach seems to be associated to a higher recurrence rate which can be up to 25%. Recurrence usually occurs during the first 2 years after the first treatment. Myxomas/MFs show a recurrence rate between 25% and 43% (6).

The aim of this work is to perform a literature review concerning epidemiology, clinical and radiographic features as well as treatment options and recurrence rates of MF.

## Material and Methods

The PubMed database was searched using the following keywords: “odontogenic myxofibroma”, “odontogenic fibromyxoma”, “myxofibroma of the jaw” and “fibromyxoma of the jaw”.

Only English articles published after 1950 were selected. Additional articles were obtained from reference lists of studies included.

Abstract from all papers retrieved were analyzed by two indipendent researchers and only cases providing details on at least gender, age and localization were selected.

## Results

Fifteen articles, reporting in details the experience with 24 patients, were identified and included in the present review (1,3,9,10-21). Two cases (8.3%) were peripheral MFs and 22 (91.7%) were central tumors.

Fourteen patients were females (58.3%), 10 were males (41.7%) (male/female ratio=1:1.4).

The youngest patient was 8 years old; the oldest was 71. Average age was 29,5 years.

Thirteen tumors (54.2%) were located in the mandible, ten of these (76.9%) affecting the posterior area. Eleven MFs (45.8%) were located in the maxilla, 6 (54.5%) in the anterior and 5 (45.5%) in the posterior area ( Table 1).

**Table 1:**
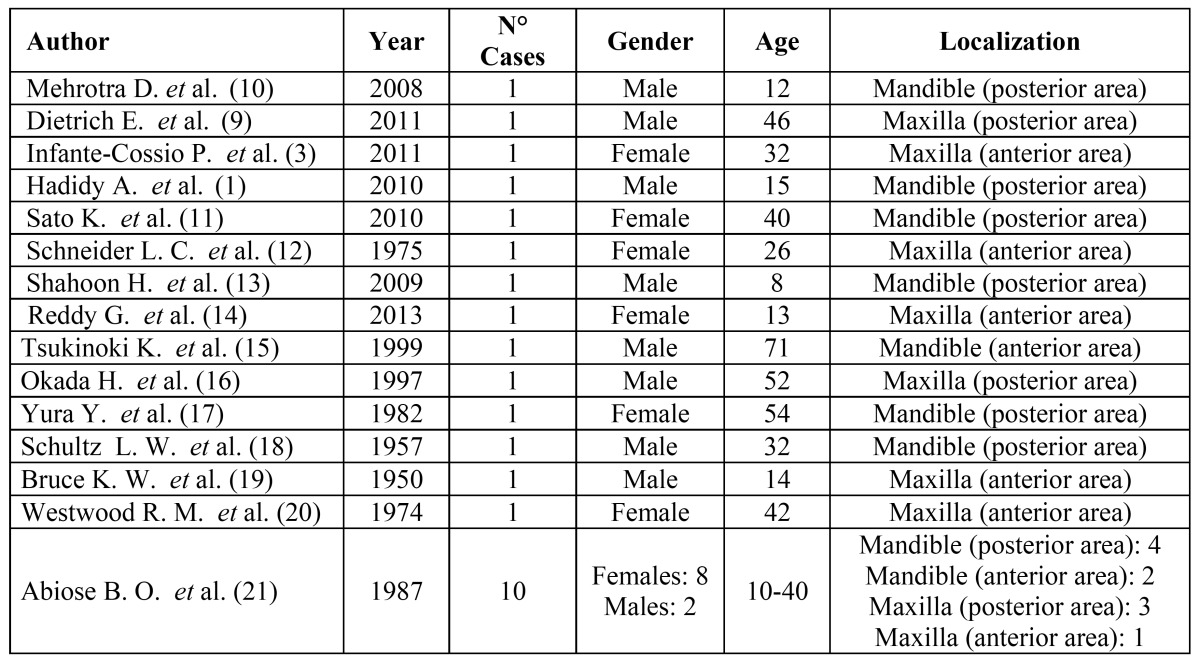
Epidemiology of odontogenic myxofibroma.

The radiographic appearances are reported in  Table 2 (1,3,9,10-21). For 10 cases no data on radiology were available.

**Table 2:**
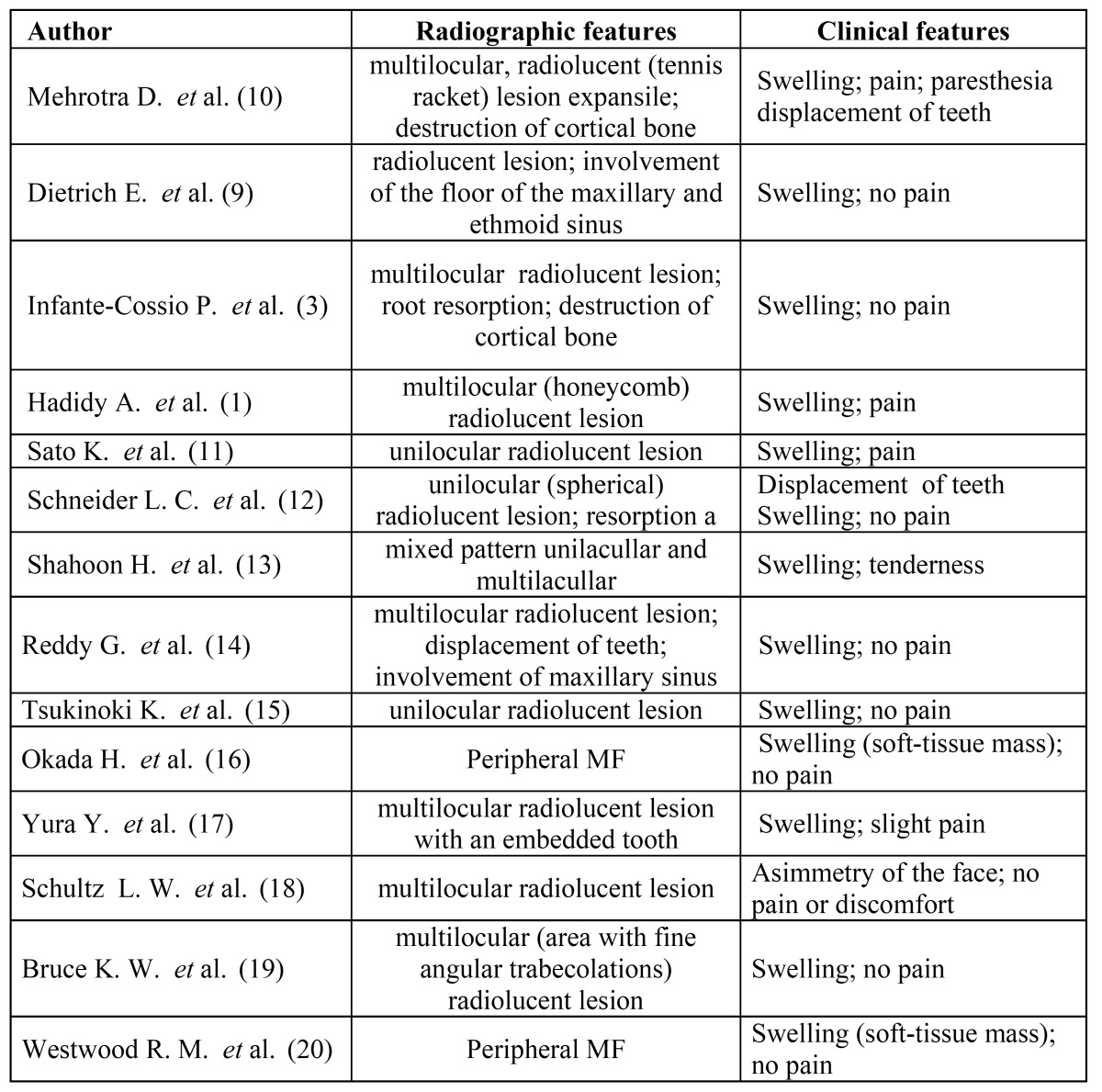
Radiographic and clinical features.

**Figure 1:**
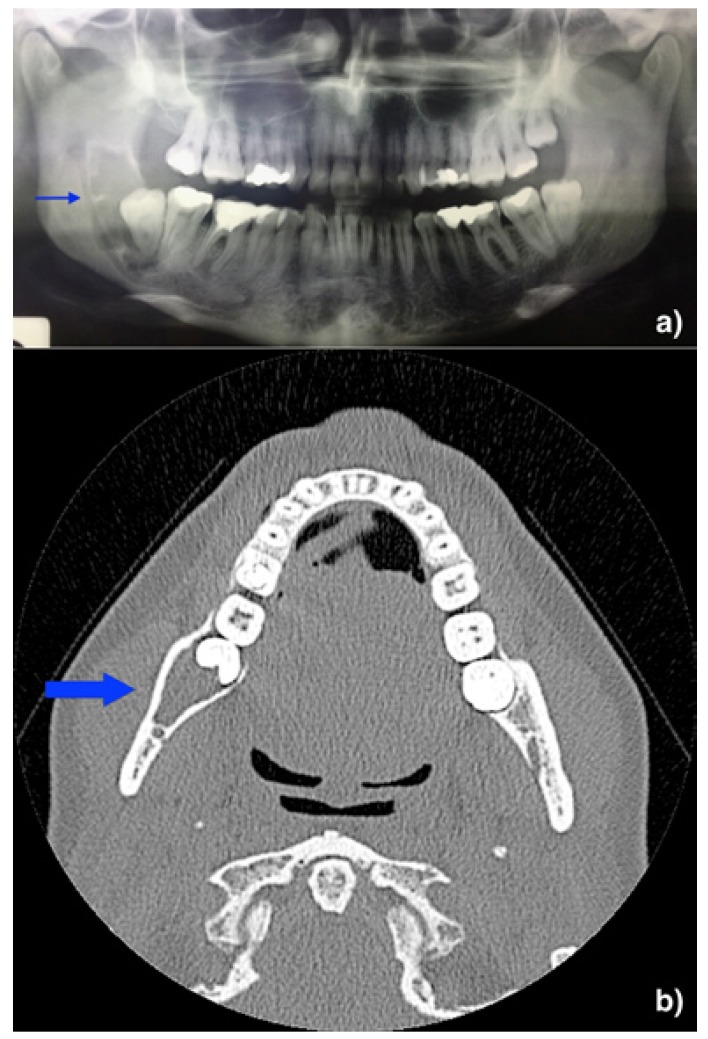
Radiographic imaging – A) orthopantomography showing a radiolucent multilocular and multilobular lesion associated with impacted right third molar of the mandible. B) axial CT Scan showing the radiolucent lesion within intact buccal and cortical bone.

The majority of cases (n=8; 66.7%) appeared as a multilocular (“tennis racket” or “honeycomb”) radiolucency; less frequently (n=4; 33.4%) MFs presented a unilocular radiographic pattern (Fig. 1).

 Table 2 shows the different signs and symptoms of 14 patients, for which data were available (1,3,9,10-21). Thirteen patients (92.86%) had swelling and five (35.71%) varying degrees of pain. Paraesthesia of the mandibular nerve was present in only one patient (7.14%). Root resorption and displacement of teeth were seen in four patients (28.57%).

**Figure 2:**
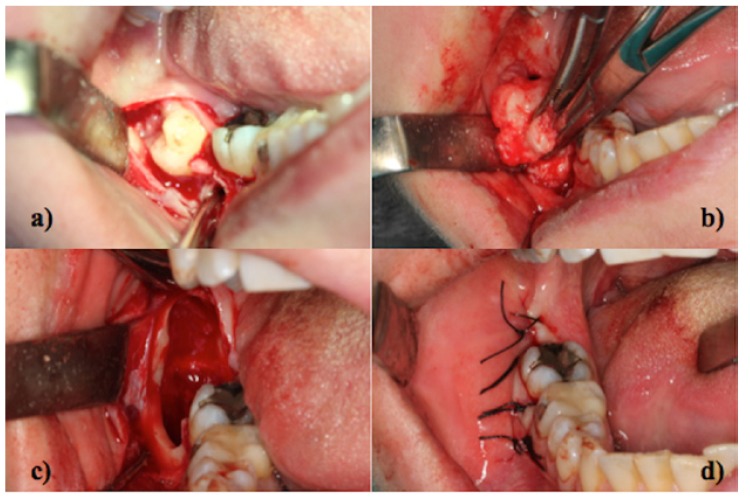
Surgical treatment – A) mucoperiosteal flap B) enucleation of the lesion C) residual cavity D) suture.

Most of the Authors reported an histologic pattern consisting of abundant myxoid connective stroma, areas of moderately dense collagen fibers, and strands of odontogenic epithelium within the connective tissue stroma (3). The tumor cells had spindle, round or stellate appearances with long anastomosing processes (11). Nuclear pleomorphism was mild and the immunohistochemical examination by means of Ki-67 labeling index revealed a low rate of cellular mitoses (9) (Fig. 2).

**Figure 3:**
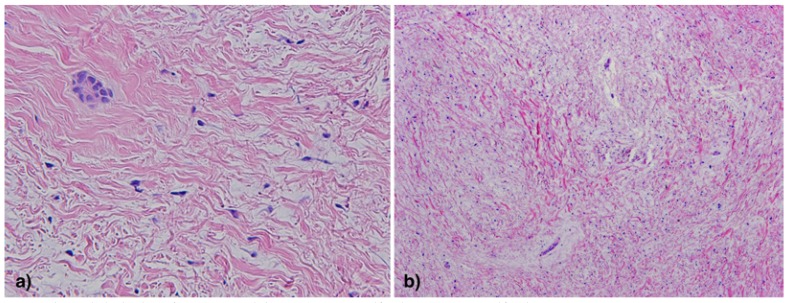
Histopathologic view of MF (H&E staining) – A) 20X magnification; B) 10X magnification.

 Table 3 shows the types of treatment and the rate of recurrence altogether with the follow-up available for each case (3,9,10-21). Six patients (26.09%) were treated with radical surgery (en-bloc resection or partial maxillectomy/mandibulectomy); seventeen (73.91%) were treated with conservative surgery (enucleation and courettage) (Fig. 3).

**Table 3:**
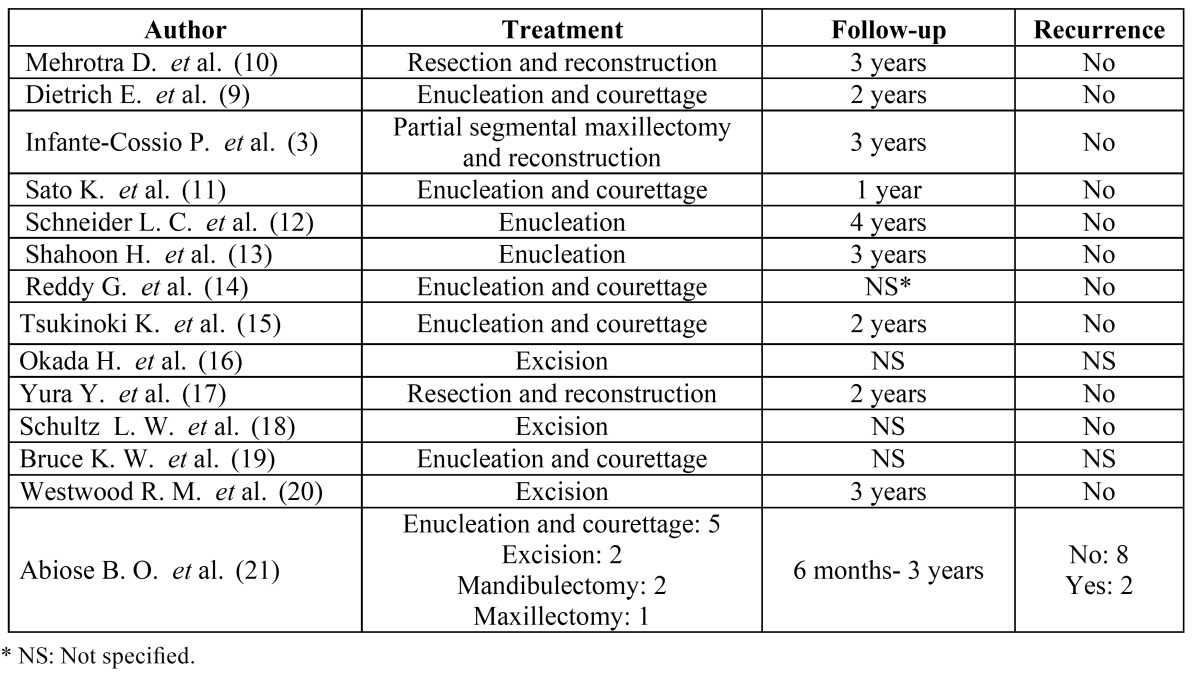
Treatment and recurrence rate.

The range of follow-up varied from 6 months to 4 years. In two out of 21 cases (9.52%) a recurrence was reported (21).

## Discussion

MFs are benign but locally aggressive odontogenic tumors which affect almost exclusively the jaws (2). The mandible is more frequently affected than the maxilla with a predilection for the posterior region in the both jaws (2). Most of the cases reported in the literature were diagnosed between the second and fourth decades of life with a peak in the third decade (2).

The majority of MFs are asymptomatic, even though few patients were reported to have increasing pain associated to invasion of surrounding structures (8). Apparently, patients with posteriorly located tumors had a late diagnosis and bigger lesions when compared to those with anteriorly located tumors. This is probably due to more visible disfigurement when the lesions are located in the anterior area.

Similarly to other odontogenic tumors (e.g. ameloblastoma), terms such as “soap-bubbles”, “ground-glass” or “tennis racquet strings” have all been used to describe the radiographic appearances of these lesions. As these descriptors may be variably interpreted and somewhat confusing, a more precise definition of the radiological features of myxomas and MFs may be warranted in an attempt to establish more objective diagnostic criteria.

According to the literature, clinical and radiographic differential diagnoses of MFs include ameloblastoma, ameloblastic fibroma, odontogenic fibroma, odontogenic keratocystic tumor, central hemangioma, aneurysmal bone cyst and other rare entities like desmoplastic fibroma. The gross appearance of the surgical specimen which displayed a hard-fibrous consistence, could lead the surgeons toward the hypothesis of a myxomatous lesion (2).

As for the other odontogenic tumors, definitive diagnosis of MFs is based on histopathologic evaluation. In case of big lesions, a biopsy may be necessary to establish the nature of the tumor and to plan the therapeutic approach. MF is not radiosensitive and the surgical excision has been reported as the treatment of choice (3). No agreement exists on the extension of surgical margins. Mainly because of the rarity of MFs, it does not seem possible to draw reliable data on prognosis after different surgical approaches. Conservative surgery, which consists on enucleation of the lesion and courettage of the residual cavity may have some advantages when compared with more radical approaches such as resection of the tumor together with some surrounding tissues. Such advantages include a reduced morbidity, the possible avoiding of reconstructive surgery, shorter hospitalization time, reduced disturbances of facial growth in children and lower costs. Nevertheless, radical treatment (e.g. en-bloc resection) is suggested by some Authors on the basis of characteristics of MFs such as the locally aggressive nature, the possible large size and tendency to recur (3,4).

Recurrence is probably associated to local invasion into cancellous bone beyond radiographically visible margins in absence of tumor encapsulation. Recurrence rate can apparently be reduced with a more aggressive treatment by performing a partial or complete segmental bone resection with tumor free margins of 1.5 cm. Such a treatment can be preferred in the maxilla, where the closeness to the maxillary sinus, the zygoma and the lower part of the orbital cavity can be a critical factor in case of recurrence (6).

The choice of conservative surgery is also supported by the absence of evidence of malignant transformation of MFs as well as the low recurrence rate reported in some case series after conservative treatment (9).

The patient should be monitored for at least three years after the surgical intervention as the recurrence rate seems to be higher during this period (6).

In conclusion, the choice of treatment mainly depends on variables such as mandibular or maxillary localization, presence of a primary or recurrent lesion, age, general medical conditions and aesthetic needs of the patient.
